# Sleep Quality, Insomnia, Daytime Sleepiness, and OSA Risk Across Occupational Groups: A Comparative Analysis of Professional Drivers and IT Workers

**DOI:** 10.3390/jcm15051860

**Published:** 2026-02-28

**Authors:** Gabriela Roxana Louisse Neacşu, Agripina Rașcu, Alexandra Beatrice Nedelcu

**Affiliations:** 1 Doctoral School, Carol Davila University of Medicine and Pharmacy, 020021 Bucharest, Romania; alexandra-beatrice.nedelcu@umfcd.ro; 2 Clinical Department 5, Faculty of General Medicine, Carol Davila University of Medicine and Pharmacy, 020021 Bucharest, Romania; 3 Department of Occupational Medicine, Colentina Clinical Hospital, 020125 Bucharest, Romania; 4 Department of Physical Medicine and Rehabilitation, Carol Davila University of Medicine and Pharmacy, 020021 Bucharest, Romania; 5 Department of Medical Assessment and Work Capacity Rehabilitation, The National Institute for Medical Assessment and Work Capacity Rehabilitation, 050659 Bucharest, Romania

**Keywords:** sleep quality, professional drivers, IT workers, insomnia, obstructive sleep apnea, occupational health, night-shift work

## Abstract

**Background:** Sleep disturbances are common in occupational settings and may impair health, safety, and work performance. Professional drivers represent a safety-critical occupational group, whereas information technology (IT) workers are frequently exposed to prolonged screen use and high cognitive workload. **Methods**: A cross-sectional study was conducted during 2023–2025 among 488 workers, including professional drivers and IT workers. Participants completed the Pittsburgh Sleep Quality Index, STOP-Bang questionnaire, Epworth Sleepiness Scale, and Athens Insomnia Scale. Age, sex, body mass index, and total work experience were recorded. Group comparisons and multivariable logistic regression analyses adjusted for demographic and occupational factors were performed. A predefined subgroup analysis was conducted among night-shift workers (*n* = 113). **Results**: IT workers were younger and reported poorer subjective sleep quality and more frequent insomnia symptoms compared with professional drivers. In contrast, moderate-to-high OSA risk was more prevalent among drivers. Excessive daytime sleepiness did not differ significantly between groups. In multivariable models, occupational group independently predicted poor sleep quality and insomnia, whereas age and body mass index were the strongest predictors of OSA risk. **Conclusions**: Sleep-related outcomes differ across occupational groups. Professional drivers appear more vulnerable to OSA-related risk, while IT workers experience a higher burden of insomnia and poor subjective sleep quality. Occupational context should be considered when designing sleep screening and prevention strategies.

## 1. Introduction

Night-shift work is associated with circadian misalignment, reduced sleep duration, and impaired physiological recovery, with potential consequences for health, safety, and work performance [1,2,3]. Disruption of the sleep–wake cycle has been linked to metabolic, cardiovascular, and neurocognitive dysfunction and represents an important occupational health concern in modern work environments [1,2].

Professional drivers constitute a safety-critical occupational group in whom sleep disturbances may have immediate implications for public safety. Excessive daytime sleepiness and untreated obstructive sleep apnea (OSA) are well-established risk factors for traffic accidents and near-miss incidents [4]. OSA prevalence is particularly high in this workforce and is strongly associated with demographic and anthropometric characteristics such as male sex and increased body mass index [5,6].

In contrast, information technology (IT) workers are typically exposed to prolonged screen use, high cognitive workload, and irregular schedules, factors that are strongly associated with poor sleep quality and insomnia symptoms [7,8,9]. Evening exposure to light-emitting devices and cognitive hyperarousal may further exacerbate sleep disturbances in this occupational group [7,8].

Despite increasing recognition that sleep-related problems differ across occupational settings, direct comparisons between distinct occupational groups exposed to night work remain limited [10,11,12,13]. In particular, few studies have simultaneously examined subjective sleep quality, insomnia symptoms, daytime sleepiness, and OSA risk in safety-critical versus cognitively demanding professions.

These considerations highlight the importance of evaluating sleep-related outcomes within their specific occupational context, including among workers exposed to night-shift schedules.

## 2. Materials and Methods

This study was designed as an original cross-sectional observational study conducted among professional drivers and IT workers. Written informed consent was obtained before enrolment.

### 2.1. Participants and Study Design

Data were collected from working adults employed as professional drivers or IT specialists between October 2023 and December 2025. The present analysis is based on primary data collected specifically for this study. Participants were recruited through occupational health assessment and workplace screening programmes. Eligibility criteria included being aged 18 years or older and having current employment as a professional driver or IT worker. Exclusion criteria included severe psychiatric or neurological disorder, known untreated sleep disorders subject to driving or occupational restrictions, and incomplete questionnaire data.

Night-shift work was defined in accordance with Article 125 of the Romanian Labour Code, which considers work performed between 10 p.m. and 6 a.m. to be night work [14]. Under this regulation, an employee is classified as a night worker if they perform at least three hours of work during this timeframe on a single working day, or if at least 30% of their monthly working hours fall within this period. Night work in Romania is subject to special regulatory protections, including limitations on daily working time and entitlement to compensatory rest or pay supplements.

A total of 488 participants (400 professional drivers and 88 IT workers) were included in the overall sample. A predefined subgroup analysis was conducted among participants engaging exclusively in night-shift work, resulting in a subsample of 113 workers (73 professional drivers and 40 IT workers).

### 2.2. Measures

All data were obtained using a standardized, self-administered questionnaire, which was completed either during the occupational health consultation or online, and in both cases, anonymously. The demographic and occupational characteristics included age, sex, BMI and total work experience (in years). The total work experience was used as an indicator of lifetime occupational exposure rather than tenure in the current role. Employees were classified as either professional drivers or IT workers. Work schedule data were used to identify employees who work night shifts.

Sleep quality was assessed using the Pittsburgh Sleep Quality Index (PSQI), which is a validated measure of perceived sleep quality over the previous month. Global scores range from 0 to 21, with higher scores indicating lower sleep quality. For categorical analyses, a PSQI global score of ≥10 was considered to indicate clinically significant poor sleep quality.

Daytime sleepiness was assessed using the Epworth Sleepiness Scale (ESS), which measures the likelihood of dozing off in everyday situations (scoring from 0 to 24). A score of ≥11 on the ESS was used to define excessive daytime sleepiness. Participants, particularly professional drivers, were informed that their responses would be kept confidential and would not be disclosed to employers or licensing authorities.

Insomnia symptoms were assessed using the Athens Insomnia Scale (AIS). Total scores (range from 0 to 24) were analyzed both continuously and categorically. Based on standard criteria, scores of 0–5 were categorized as no insomnia, 6–9 as mild insomnia, 10–15 as moderate insomnia, and 16 or above as severe insomnia. For the primary analysis, a cut-off point of AIS ≥ 6 was used to define clinical insomnia.

The risk of OSA was estimated using the STOP-Bang questionnaire, which incorporates symptoms such as snoring and daytime tiredness, as well as hypertension and anthropometric risk factors. Scores were categorized as low, intermediate, or high OSA risk. For the regression analyses, a binary variable representing a high risk of OSA was also computed, defined as a STOP-Bang score of 3 points or higher.

### 2.3. Statistical Analysis

Statistical analyses were performed to address the predefined study objectives using Jamovi software (version 2.6; The Jamovi Project, Sydney, Australia) [15].

Continuous variables were inspected for distributional properties and summarized as mean ± standard deviation, while categorical variables were presented as frequencies and percentages. Group differences between professional drivers and IT workers were examined using independent sample *t*-tests for continuous variables and chi-squared tests for categorical variables. Effect sizes were reported as Cohen’s d and Cramer’s V, respectively.

The primary analyses focused on sleep quality (PSQI), insomnia severity (AIS), EDS, and OSA risk (STOP-Bang), which were examined as both continuous variables and binary outcomes using clinically established thresholds. Additional subgroup analyses were performed among participants engaged exclusively in night shift work.

Binary logistic regression analyses were conducted to identify the independent predictors of poor sleep quality (PSQI ≥ 10), clinical insomnia (AIS ≥ 6) and high OSA risk (STOP-Bang ≥ 3). Binary predictors were coded as sex (female = 0, male = 1) and occupation (driver = 0, IT worker = 1). Continuous predictors included age (years), BMI (kg/m^2^) and total work experience (years). The results are reported as odds ratios (OR) with 95% confidence intervals (CIs). An OR greater than 1 indicates a higher risk among males compared with females, and among IT workers compared with drivers. Model fit was evaluated using deviance statistics and pseudo-R^2^ indices. A two-tailed *p*-value of less than 0.05 was considered statistically significant.

## 3. Results

**Figure 1:**
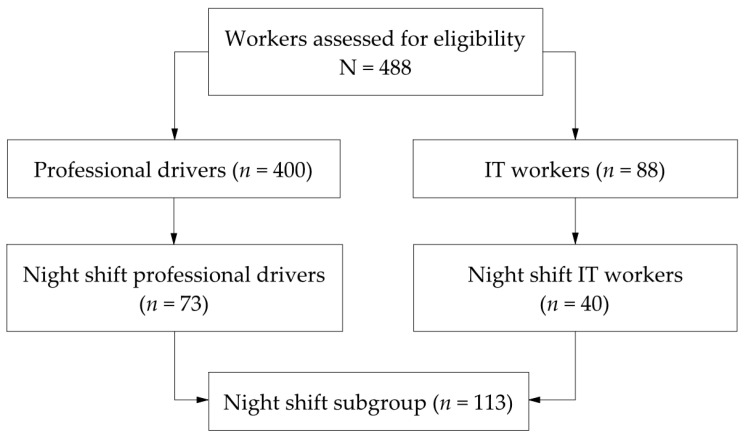
Flow diagram of the study population.

A total of 488 workers were included in the final analysis, comprising 400 professional drivers and 88 IT workers. A predefined subgroup analysis was conducted among participants engaged exclusively in night-shift work (*n* = 113; 73 professional drivers and 40 IT workers) as illustrated in Figure 1.

### 3.1. Participant Characteristics

Of the 488 participants, 400 (82%) were professional drivers, while 88 (18%) were IT workers. The mean age of the entire sample was 46.7 ± 11.9 years. Drivers were significantly older than IT workers (51.3 ± 8.6 vs. 32.5 ± 6.8 years; *p* < 0.001). The majority of drivers were male, whereas the IT group included a higher proportion of females (see Table 1). Drivers have a significantly higher body mass index and demonstrated a greater prevalence of overweight and obesity than IT workers. Total work experience was substantially higher among drivers, reflecting their older age distribution. A predefined subgroup analysis was conducted among 113 participants who reported regular night shift work, consisting of 73 drivers and 40 IT workers. Poor sleep quality was significantly more prevalent among IT workers than drivers (65.9% vs. 22.0%; χ^2^ = 66.3; *p* < 0.001, Cramer’s V = 0.37). Professional drivers exhibited a higher prevalence of intermediate to high STOP-Bang risk than IT workers (52.0% vs. 26.1%; χ^2^ = 19.8; *p* < 0.001; Cramer’s V = 0.20). Clinically significant insomnia symptoms were more prevalent among IT workers (54.5% vs. 10.7%; χ^2^ = 101; *p* < 0.001; Cramer’s V = 0.46). Overall, ESS remained uncommon, but was more prevalent among IT workers (8.0% vs. 2.5%; χ^2^ = 6.38; *p* = 0.012).

### 3.2. Sleep Quality (PSQI)

There were significant differences in sleep quality between occupational groups. The mean PSQI score was higher among IT workers than among drivers (10.9 ± 3.9 vs. 8.3 ± 3.6; *p* < 0.001). Using the PSQI clinical cut-off score of 10, 70% of IT workers and 30% of drivers met the criteria of poor sleep quality that is considered clinically relevant (χ^2^ = 32.4; *p* < 0.001; Cramer’s V = 0.536), representing a large effect size. This pattern remained consistent in the night-shift-only subgroup.

### 3.3. Insomnia Symptoms (AIS)

Insomnia severity followed a similar pattern. IT workers recorded significantly higher AIS scores than Drivers. Clinical insomnia (AIS ≥ 6) was observed in 44.3% of IT workers, compared to 14.5% of drivers. This corresponds to a prevalence of insomnia-related symptoms that is more than three times higher in IT workers (χ^2^ = 31.1; *p* < 0.001; Cramer’s V = 0.524). Again, the difference remained evident in the night-shift-only subgroup (see Table 2).

### 3.4. Risk of OSA (STOP-Bang)

Unlike subjective sleep disturbance, the risk of OSA was significantly higher among professional drivers. A STOP-Bang score of at least 3 was identified in 79.5% of drivers and 20.5% of IT workers (χ^2^ = 7.04; *p* = 0.008, Cramer’s V = 0.250). This difference remained consistent after adjusting for age and BMI in regression analyses.

### 3.5. Daytime Sleepiness (EDS)

Excessive daytime sleepiness was uncommon in the overall sample.

EDS was identified in 3.5% of the total sample. Within the full cohort, ESS scores of 11 or above were more prevalent among IT workers than among professional drivers (8% vs. 2.5%; *p* = 0.012). However, within the night-shift-only subgroup, the overall prevalence was 7.1%, with no significant difference observed between the IT and professional driver groups (7.5% vs. 6.8%; *p* = 0.897) (Table 2).

### 3.6. Multivariable Regression Analysis

Binary logistic regression analysis showed that occupational group (IT work) was an independent predictor for both poor sleep quality (PSQI ≥ 10) and insomnia (AIS ≥ 6), even after adjusting for age, sex, BMI and total work experience (Table 3A). Male sex was also found to be an independent predictor of PSQI scores of 10 or more. However, age and BMI were found to be the strongest predictors of increased OSA risk, while occupational group was not independently associated with a high-risk STOP-Bang classification once these factors were considered. No predictors were independently associated with excessive daytime sleepiness (Table 3B).

**Figure 2:**
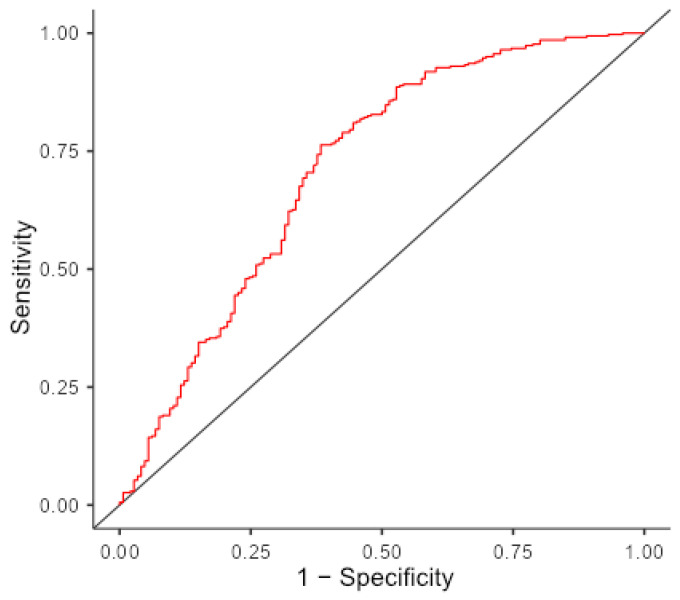
ROC Curve for the Logistic Regression Model Predicting PSQI ≥ 10. The red curve represents the model’s discriminatory performance, and the diagonal line indicates the line of no discrimination (AUC = 0.5).

The multivariable model predicting PSQI ≥ 10 showed acceptable discriminatory ability, with an area under the ROC curve of 0.713 (Figure 2). Model diagnostics indicated an acceptable fit and stability across analyses.

## 4. Discussion

### 4.1. Principal Findings

This study compared sleep quality, insomnia symptoms, daytime sleepiness, and OSA risk between professional drivers and IT workers. It also examined a predefined subgroup of employees engaged in night shift work. The main finding was that IT workers reported significantly lower subjective sleep quality and higher insomnia severity than drivers. However, professional drivers demonstrated a substantially greater risk profile for OSA. These results suggest that the occupational context in which sleep disturbance occurs may influence its manifestations, even among populations exposed to similar circadian disruption through night work.

The high prevalence of OSA risk observed among drivers in this study is consistent with recent data showing that professional truck drivers have a substantially higher prevalence of OSA than the general population. Prevalence estimates range from 28–40%, with screening-based high-risk rates reaching 70% in some cohorts [16,17]. This excess risk is largely driven by the typical demographic and anthropometric features of this workforce, such as being middle-aged, male and having an elevated BMI.

Untreated OSA and related sleep debt have been consistently linked to impaired attention and vigilance deficits, as well as an increased risk of crashes in safety-critical occupation such as transport [4]. These findings reinforce the importance of systematic OSA screening and treatment among driver fitness-to-work programmes, particularly as many affected individuals remain undiagnosed in routine occupational practice.

In contrast, IT workers demonstrated significantly higher levels of subjective sleep disturbance and insomnia symptoms. These findings align with existing evidence indicating a high prevalence of poor sleep quality among IT professionals, which has been associated with occupational factors such as cognitive workload, screen exposure, and work-related stress. For instance, recent cross-sectional studies have reported that a significant portion of IT professionals exhibit poor sleep quality, as measured by the PSQI, and have identified substantial associations between sleep disturbance, caffeine consumption, workstation characteristics and work demands in this group [18,19].

Experimental evidence demonstrates that evening exposure to light-emitting screens suppresses melatonin secretion and delays circadian timing, leading to prolonged sleep-onset latency and reduced total sleep duration [7]. These mechanisms are particularly relevant for occupations characterized by prolonged screen use, such as IT workers.

In addition, cognitive hyperarousal and persistent pre-sleep rumination are recognized as core mechanisms in the pathophysiology of insomnia, particularly in individuals exposed to high cognitive demands and a sustained mental workload [8]. These processes may be particularly pertinent to younger professional groups engaged in cognitively intense work, such as IT workers, and may partly account for the higher prevalence of insomnia-type sleep disturbances observed in this group.

Interestingly, the prevalence of EDS was relatively low overall. Among drivers, reported sleepiness was lower than expected given the observed OSA risk profile. Previous studies have shown that subjective sleepiness measures alone may underestimate the true burden of fatigue in commercial drivers [4]. This discrepancy may reflect symptom under-reporting in safety-critical occupations where excessive sleepiness has legal or occupational consequences. In contrast, IT workers, who are not subject to licensing restrictions, may be more open about their symptoms. These findings emphasize the limitation of relying exclusively on self-reported daytime sleepiness measures in occupational screening.

OSA is more prevalent among professional drivers and has been consistently linked to an increased risk of crashes and adverse safety outcomes when appropriate screening and treatment are lacking [20].

### 4.2. Clinical and Occupational Implications

From an occupational health perspective, the findings suggest the need for tailored sleep health strategies. For professional drivers, the focus should be on the early identification and treatment of OSA and associated cardiometabolic risk. This should be supported by systems that do not penalize the reporting of symptoms, to reduce fear-driven under-disclosure. For IT workers, behavioral sleep interventions, digital hygiene education and access to cognitive therapy for insomnia may be particularly appropriate given their younger age distribution and insomnia-dominant symptom profile.

### 4.3. Strengths and Limitations

The marked numerical and demographic differences between professional drivers and IT workers represent an important limitation of this study. Although multivariable analyses were adjusted for age, sex, body mass index, and work experience, residual confounding related to group imbalance cannot be fully excluded. Therefore, the observed differences should be interpreted as occupation-related patterns rather than direct causal effects.

Sex distribution differed substantially between groups, with a higher proportion of women among IT workers. Given that female sex has been consistently associated with higher rates of insomnia and poorer subjective sleep quality, sex represents an important potential confounder. Although sex was included as a covariate in multivariable analyses, its influence on subjective sleep outcomes should be considered when interpreting the findings.

A major limitation of this study relates to the reliance on self-reported sleepiness measures among professional drivers. Given the legal and occupational consequences associated with excessive daytime sleepiness in this group, under-reporting of symptoms—particularly on the Epworth Sleepiness Scale—cannot be excluded. The combination of a high risk of OSA and relatively low reported daytime sleepiness among drivers supports this concern and suggests that subjective sleepiness questionnaires may underestimate true functional impairment in safety-critical occupations.

Additional limitations should be acknowledged. Seasonal variation in natural light exposure, which may influence circadian entrainment and sleep outcomes, was not systematically accounted for. Chronotype and social jet lag—important modifiers of individual tolerance to shift work—were not assessed, as chronobiological questionnaires were not included in the original study protocol. In addition, information regarding prior SARS-CoV-2 infection was not available, although emerging evidence suggests potential long-term effects on sleep and circadian regulation. These factors should be considered in future studies aiming to further refine occupational sleep risk assessment.

The strengths of this study include two distinct occupational groups undergoing real-world screening, the use of validated sleep questionnaires, and the analysis of a predefined night-shift subgroup. Nevertheless, the cross-sectional design precludes causal inference, and objective sleep assessments were not available for the entire study population. Although respiratory polygraphy was performed on a subset of professional drivers as part of their routine occupational health evaluation, objective sleep recordings were not systematically collected for all participants and were therefore not included in the present analyses.

### 4.4. Overall Interpretation

Overall, this study shows that sleep vulnerability varies depending on the occupation, rather than being uniform across night-shift workers. Professional drivers appear to be particularly vulnerable to OSA-related risk, whereas IT workers demonstrate a higher burden of insomnia-related disturbance. It is essential to recognize the heterogeneity when designing targeted prevention and intervention strategies.

## 5. Conclusions

This study demonstrates that sleep disturbance across occupational groups is not uniform, including among workers exposed to night-shift schedules, and varies according to occupational context. Professional drivers were substantially more likely to meet the criteria for elevated OSA risk, whereas IT workers experienced disproportionately higher rates of poor subjective sleep quality and insomnia symptoms, despite being younger and having a lower BMI. These findings suggest that different patterns of sleep disturbances may be present across occupational groups. The observed risk profile among drivers is consistent with mechanisms commonly associated with OSA. Among IT workers, however, the predominance of insomnia-type symptoms may reflect circadian and behavioral factors.

These findings underscore the value of directly comparing distinct occupational groups and support the use of occupation-specific approaches when evaluating sleep-related outcomes, including in night-shift workers.

From an occupational health perspective, these results highlight the importance of occupation-specific sleep screening and prevention strategies, rather than uniform approaches applied across diverse occupational groups.

Overall, the results support moving away from the generic concept of shift work sleep problems towards occupational-specific prevention and management approaches. However, future longitudinal studies incorporating objective sleep measures are needed to clarify causality and long-term clinical outcomes.

## Figures and Tables

**Table 1:** Baseline demographic and occupational characteristics of professional drivers and IT workers.

Variable	Drivers (*n* = 400)	IT Workers (*n* = 88)	*p*-Value	Effect Size
Age, years				
mean ± SD	51.1 ± 5.76	32.5 ± 11.8	<0.001	Cohen’s d = 2.57
median, IQR	52.0 (47–56)	30.0 (24–38)		
Total work experience, years				
mean ± SD	29.7 ± 7.40	10.2 ± 9.61	<0.001	Cohen’s d = 2.50
median, IQR	30.0 (25–35)	7.0 (3–18)		
BMI, kg/m^2^				
mean ± SD	31.5 ± 5.47	26.6 ± 5.85	<0.001	Cohen’s d = 0.89
median, IQR	31.0 (28–34)	25.4 (22–29)		
Female sex, *n* (%)	19 (4.8%)	45 (51.1%)	<0.001	Cramer’s V ≈ 0.40

Continuous variables are expressed as the mean ± standard deviation or the median (interquartile range). Group comparisons were performed using independent-sample *t*-tests with Welch correction where necessary, as well as chi-square tests for categorical variables.

**Table 2:** Comparison of sleep-related outcomes between professional drivers and IT workers.

Sleep Outcome	Professional Drivers (*n* = 400)	IT Workers (*n* = 88)	χ^2^	*p*-Value	Effect Size (Cramer’s V)
PSQI ≥ 10 (poor sleep), *n* (%)	88 (22.0%)	58 (65.9%)	66.3	<0.001	0.369
STOP-Bang risk category, *n* (%)			19.8	<0.001	0.201
Low risk	192 (48.0%)	65 (73.9%)			
Intermediate risk	145 (36.3%)	18 (20.5%)			
High risk	63 (15.8%)	5 (5.7%)			
Insomnia severity (AIS), *n* (%)			101	<0.001	0.455
No insomnia	357 (89.3%)	40 (45.5%)			
Mild insomnia	34 (8.5%)	27 (30.7%)			
Moderate insomnia	8 (2.0%)	16 (18.2%)			
Severe insomnia	1 (0.2%)	5 (5.7%)			
ESS ≥ 11 (excessive daytime sleepiness), *n* (%)	10 (2.5%)	7 (8.0%)	6.38	0.012	0.114

Data are presented as numbers (percentages). Group differences were analyzed using chi-square tests. The effect size was reported using Cramer’s V. A *p*-value of less than 0.05 was considered statistically significant.

**Table 3:** (**A**) Multivariable logistic regression analysis of predictors of poor sleep quality and insomnia. (**B**) Multivariable logistic regression analysis of predictors of increased OSA risk and excessive daytime sleepiness.

**(A)**				
**Predictor**	**PSQI ≥ 10 OR (95% CI)**	***p*-Value**	**AIS ≥ 6 OR (95% CI)**	***p*-Value**
Occupation: Driver (ref = IT)	0.20 (0.09–0.44)	<0.001	3.06 (1.20–7.80)	0.02
Male sex (ref = female)	3.58 (1.83–7.00)	<0.001	0.38 (0.19–0.75)	0.006
Age (years)	1.05 (0.99–1.12)	0.081	1.03 (0.98–1.07)	0.211
Total employment (years)	0.95 (0.90–1.00)	0.056	1.00 (0.94–1.06)	0.924
BMI (kg/m^2^)	1.01 (0.95–1.07)	0.742	1.03 (0.98–1.08)	0.211
**(B)**				
**Predictor**	**STOP-BANG ≥ 3 OR (95% CI)**	***p*-Value**	**ESS ≥ 11 OR (95% CI)**	***p*-Value**
Occupation: Driver (ref = IT)	3.06 (1.20–7.80)	0.02	1.35 (0.24–7.63)	0.736
Male sex (ref = female)	2.01 (0.84–4.83)	0.118	2.08 (0.65–6.84)	0.191
Age (years)	1.10 (1.04–1.17)	0.001	1.03 (0.93–1.13)	0.602
Total employment (years)	0.99 (0.94–1.04)	0.577	0.93 (0.84–1.02)	0.124
BMI (kg/m^2^)	1.26 (1.20–1.33)	<0.001	1.04 (0.96–1.13)	0.351

(OR = odds ratio; CI = confidence interval; Reference groups: IT workers and female sex; PSQI ≥ 10 indicates poor sleep quality; AIS ≥ 6 defines clinical insomnia; ESS ≥ 11 defines excessive daytime sleepiness; STOP-Bang ≥ 3 indicates increased OSA risk. All models adjusted for occupation, sex, BMI, age and total employment duration).

## Data Availability

The data presented in this study are available on reasonable request from the corresponding author due to privacy and ethical restrictions.
